# Constructing an indicator system for factors influencing volleyball coaches' in-game tactical decision-making: a Delphi study

**DOI:** 10.3389/fpsyg.2025.1721316

**Published:** 2026-01-12

**Authors:** Zheng Sun, Fan Zhang

**Affiliations:** 1 School of Physical Education, Anhui Normal University, Wuhu, China; 2 Department of Kinesiology & Health Promotion, University of Kentucky, Lexington, KY, United States

**Keywords:** Delphi method, indicator system, influencing factors, in-game tactical decision-making, volleyball coaches

## Abstract

The purpose of this study was to construct a systematic and evidence-based framework of indicators for factors influencing volleyball coaches' in-game tactical decision-making. Potential indicators were generated through expert screening and group discussions, then refined via two rounds of Delphi consultation. Indicator weights were determined using the precedence chart method. The final framework comprised six primary indicators—Athlete Competitive Performance (0.3056), Coach Competence and On-site Performance (0.2500), Match Situation and Dynamics (0.1944), Information Support (0.1389), Officiating Performance (0.0566), and Spectator Behavior and Unexpected Incidents (0.0566)—along with 15 secondary and 52 tertiary indicators. Athlete Competitive Performance carried the greatest weight, while Officiating Performance and Spectator Behavior showed the lowest. At the secondary level, Coach Competence, Team Coordination, and both Own and Opponent Athlete Performance were the most influential factors. At the tertiary level, the Individual Technical and Tactical Performance of Own Players was most critical, followed by tactical execution, lineup strength, coaches' authority, and opponent tactical adjustments. Overall, the study highlights the complexity of in-game tactical decision-making and provides a structured indicator framework that offers theoretical insights and practical guidance for improving rational and adaptive coaching decisions in competitive volleyball.

## 1 Introduction

In modern volleyball, tactical decision-making by coaches has become a decisive factor beyond the athletic contest among players. This influence is particularly evident when teams are evenly matched, as the coach's in-game decisions can directly shape the course and outcome of the match. Volleyball can be seen as a complex system comprising players, coaches, referees, and spectators, with the confrontation between athletes at its core. The actions of other elements within this system are driven by this central dynamic, generating both challenges and opportunities. In-game tactical decision-making, therefore, is the process by which coaches coordinate these confrontations, address emerging problems, and capitalize on opportunities, producing new methods, strategies, and outcomes. Essentially, it is a cognitive activity centered on judgment and choice, characterized by immediacy and situational specificity (Costa et al., 2018).

As the leaders of competition, volleyball coaches rely on tactical decisions to enable their teams to act effectively within contexts of constraint and counter-constraint, thereby achieving desired goals. Such decisions must be grounded in accurate understanding and scientific analysis of multiple subjective and objective factors, as well as their interrelationships. Previous studies have indicated that systematic performance analysis provides coaches with valuable informational support in dynamic competition environments, thereby improving the scientific rigor and effectiveness of their tactical decision-making (Lorains et al., 2013). Moreover, contextual variables, such as the competition environment and team status, also influence coaches' perceptions of player performance and their tactical adjustments during the match (López-Serrano et al., 2022). Thus, Constructing an indicator system of influencing factors can therefore help coaches analyze these conditions systematically and formulate appropriate strategies to accomplish competitive objectives.

Coaches' tactical decision-making is a complex cognitive process, deeply influenced by various multidimensional situational factors. A review of the literature reveals that research on the factors affecting coaches' tactical decision-making is often embedded in broader studies on on-site coaching and situational decision-making, primarily focusing on the following four aspects.

### 1.1 Coaches' personal competence

Coaches' personal competence provides the foundational cognitive framework and psychological support for their tactical decision-making, covering professional skills, knowledge structure, experience, psychological factors, and authority. The scientific nature of decision-making relies on a coach's professional skills and knowledge structure. Costa et al. (2018) pointed out that a coach's expertise enables them to quickly identify and respond to complex situations during matches. Vergeer and Lyle (2009) emphasized that a coach's knowledge structure not only includes an understanding of techniques and tactics but also extends to a comprehensive understanding of athletes' abilities and match contexts, which significantly influences tactical adjustments. Moreover, extensive on-site experience provides valuable intuitive decision-making support, helping coaches make quick and effective responses in complex situations (Roca and Gomes, 2022; Raab, 2003).

In match contexts, coaches often face cognitive overload and psychological stress (Fletcher and Scott, 2010), which can lead to suboptimal decisions. Kaya (2014) argued that the multidimensional nature of decision-making in complex environments requires coaches to possess effective psychological coping abilities. Zajonz et al. (2024) further found that coaches with high emotional intelligence are better able to remain calm under pressure, preventing emotional fluctuations from interfering with decision-making, leading to more rational judgments (Zajonz et al., 2024). Additionally, Rylander (2016) suggested that in team sports, coaches can influence the behavior of team members through their power base (e.g., professional authority, leadership influence), thereby improving team performance and coaching effectiveness. These factors all influence coaches' tactical decision-making to varying degrees.

### 1.2 Coaches' leadership styles and behaviors

Coaches' leadership styles and behaviors significantly impact the appropriateness and execution of their tactical decisions. Jawoosh et al. (2022) found that a coach's leadership style significantly affects athlete satisfaction and team collaboration, with transformational leadership styles stimulating athletes' enthusiasm and creativity, while authoritarian styles may suppress athletes' initiative. Jin et al. (2022) further indicated that leadership style affects the coach-athlete relationship, enhancing athlete motivation and satisfaction, which in turn facilitates tactical decision execution. Furthermore, Oh (2023) argued that effective communication by the coach and high team cohesion amplify the positive effects of transformational leadership, improving athlete performance and optimizing tactical execution. Coaches' leadership behaviors are key factors influencing athletes' psychological resilience and competitive performance (Wang, 2023). Liu et al. (2025) further suggested that coaches' leadership behaviors, by improving the coach-athlete relationship, help alleviate athletes' psychological fatigue, thereby enhancing their tactical execution capabilities.

### 1.3 Match environmental factors

Various factors in the match environment (such as altitude, weather changes, audience support, and referee decisions) also directly or indirectly influence coaches‘ tactical decision-making. Aughey et al. (2013) found that high-altitude environments result in decreased performance among athletes from lower altitudes, leading to reduced running distances. Extreme heat and heavy rainfall significantly affect athletes' physical and cognitive performance (Yuan et al., 2024), resulting in diminished athletic output. Referee performance and audience emotional responses can influence athletes‘ psychological states, indirectly affecting the effectiveness of tactical execution. Catteeuw et al. (2009) noted that factors such as referees' years of experience, weekly training hours, and match officiating frequency are positively correlated with officiating skills. Furthermore, good communication and collaboration between referees can improve officiating quality (Diotaiuti et al., 2017). However, biases in refereeing still exist in real-world competitions. Avugos (2024) highlighted that referee bias could affect the fairness of decisions, indirectly influencing the effectiveness of tactical arrangements. In addition, audience members often display strong emotional biases when watching matches. Wolfson et al. (2005) found that home-court support from the audience is one of the most significant advantageous factors, although this advantage can fluctuate during the match (Marcelino et al., 2009). Intense emotional reactions and behaviors from the audience can affect athletes' psychological states, influencing the effectiveness of tactical decision execution (Bilalić et al., 2021; Oldfield et al., 2024). For example, noise (e.g., shouting, booing, cheering, verbal abuse, and musical instruments) not only affects athletes' physiological and psychological responses (Greer, 1983), but can also interfere with referee decisions (Nevill et al., 2002).

### 1.4 Team performance and athlete conditions

Coaches' tactical decisions are directly influenced by the competitive performance of both teams, athletes' conditions, injuries, and other related factors. In competitive sports, match analysis is one of the primary methods for collecting data on team and athlete performance (Fernandez-Echeverria et al., 2017), aiming to identify key indicators that are highly correlated with match outcomes, thus increasing the chances of victory (Peña and Casals, 2016; Giatsis, 2023). These studies indirectly demonstrate that the performance of athletes during matches is a core factor influencing match outcomes. With the rapid development of artificial intelligence and big data technologies, coaches have easier access to collect data on team performance (McGillick et al., 2024). This data provides valuable support for coaches' tactical decision-making. Suárez et al. (2020) found that when formulating tactics, coaches not only need to evaluate athletes' technical performance but also consider their emotional fluctuations and psychological pressures, especially when working with adolescent athletes. These factors significantly affect the execution of tactics (Suárez et al., 2020). Additionally, athletes' neural and muscular fatigue directly influence tactical decisions. In high-intensity matches, mental and muscular fatigue can impair athletes' reaction times and precision in movement, thus affecting tactical execution and overall performance (Coutinho et al., 2018). Athletes' injury conditions also impact tactical adjustments. If a key player is injured, coaches need to adjust the lineup or tactical strategies to accommodate personnel changes or the athlete's physical condition (Surya et al., 2015).

Previous studies provide valuable insights, but also reveal issues such as fragmented research content and limitations in research perspectives. For example, many studies focus on isolated or partial factors, or adopt a single theoretical perspective, failing to integrate them into a unified and comprehensive indicator system. As a result, a holistic framework for understanding and evaluating the multiple, interacting elements that influence coaches' tactical decision-making during competition is still lacking.

In response, this study adopts a systemic analytical framework to identify and refine factors influencing volleyball coaches' in-game tactical decision-making and construct an indicator system. The goal is to clarify what factors affect coaches' decisions during competition and to assess their relative importance, thereby offering theoretical grounding and data support for practical coaching strategies.

## 2 Research methods

### 2.1 Delphi method

The Delphi method is a structured technique for achieving expert consensus. It involves inviting experts from relevant fields to participate in anonymous, multi-round surveys. After each round, the experts' opinions are summarized and anonymously fed back to them. This allows experts to adjust or confirm their judgments based on group feedback until a consensus is reached or the majority opinion stabilizes (Shang, 2023). Through its mechanisms of anonymity, multi-round feedback, and statistical aggregation, the Delphi method has been widely applied in health sciences and sports research. It is particularly useful in the development of evaluation systems, decision-making standards, and consensus frameworks. Examples include physical fitness assessments, behavior report cards, and sports intervention standards (Ye et al., 2024; Chen et al., 2024).

In this study, we employed the Delphi method to construct a tactical decision-making indicator system for volleyball coaches. This method effectively integrates expert experience and judgment, making it especially suitable for situations where empirical data are insufficient, and decision-making factors are complex and heavily reliant on expert subjective experience.

#### 2.1.1 Expert panel composition

There is no unified consensus in the existing research regarding the number of experts to consult. Some studies suggest that in Delphi research involving interdisciplinary, multi-field experts or multiple stakeholders, a panel size of 60–80 participants is considered reasonable and can provide high replicability (Manyara et al., 2024). However, in Delphi research focusing on a single field, the most common expert panel sizes typically range from 8 participants (Paul, 2008) to 20 participants (Jorm, 2015). Additionally, some studies suggest that the Delphi expert panel size should be determined based on time and financial constraints, with an ideal size of 8 to 23 participants, a range that is considered practical in real-world applications (Shang, 2023).

Due to time and financial constraints, 15 experts and coaches were invited. However, three were unable to participate due to work or competition commitments, resulting in a final panel of 12 experts (Table 1). The panel comprised university scholars specializing in volleyball research and head coaches of provincial or higher-level teams. Eligibility criteria required substantial academic contributions or more than 10 years of practical experience, an associate senior or higher academic rank, or a senior or above coaching qualification. Participants were expected to be familiar with volleyball competition and tactical decision-making and to demonstrate active willingness to engage in the study. The panel included six university professors with recognized expertise and academic reputation in volleyball research, as well as six active head coaches of provincial or higher-level teams, all of whom held senior coaching qualifications and had extensive experience in match management and tactical decision-making.

**Table 1:** Basic information on consulting experts (*N* = 12).

**Number**	**Gender**	**Title**	**Research or area of expertise**
1	Male	Professor	Volleyball performance analysis
2	Male	Professor	Basic theories of Volleyball and teaching & training
3	Male	Professor	Volleyball theory and practice
4	Male	Professor	Volleyball teaching & training
5	Male	Professor	Volleyball teaching & training
6	Male	Professor	Volleyball teaching & training
7	Male	Senior coach	Head coach
8	Male	National-level coach	Head coach
9	Male	National-level coach	Head coach
10	Male	Senior coach	Head coach
11	Male	National-level coach	Head coach
12	Male	National-level coach	Head coach

#### 2.1.2 Consultation methods and procedures

After screening expert experience and conducting group discussions, a Consultation Questionnaire for Constructing an Indicator System of Influencing Factors in Volleyball Coaches' In-Game Tactical Decision-Making was developed. The questionnaire used a five-point Likert scale (1 to 5) to assess the perceived importance of each factor. Two rounds of expert consultation were conducted.

In the first round, the questionnaire was distributed via email and WeChat. The main objectives were to gather background information on the experts, evaluate their authority, gather feedback on the structure and content of the indicator system, and obtain importance ratings for the indicators.

In the second round, responses from the first round were analyzed. Based on expert feedback, the indicator system was revised, and descriptive statistics (including maximum, minimum, mean values, and percentage distributions) for each score were calculated. These results, along with the initial ratings from individual experts and the consolidated opinions of all participants, were included in the second-round questionnaire for further consultation and validation.

#### 2.1.3 Expert engagement, authority, and consensus

Two rounds of expert consultation were conducted in this study. In the first round, 15 questionnaires were distributed, 12 were returned, and all were valid, resulting in response and validity rates of 80%. In the second round, after revising the questionnaire based on the results and feedback from the first round, 12 questionnaires were distributed, all of which were returned and valid, yielding response and validity rates of 100%. These outcomes indicate that the experts demonstrated strong engagement and high enthusiasm for the study.

Analysis of authority revealed that the mean judgment basis coefficient was 0.94, the mean familiarity coefficient was 0.85, and the overall authority coefficient (Cr) was 0.895. This suggests that the experts possessed a high level of authority, were familiar with the research domain, and provided reliable opinions and suggestions.

The degree of consensus among experts was assessed using the coefficient of variation (Cv) and Kendall's coefficient of concordance (W). The coefficient of variation (Cv) is a measure of the data's dispersion, defined as the ratio of the standard deviation to the mean. A higher Cv value indicates greater variability in the data. Kendall's coefficient of concordance (W) is used to assess the level of agreement among multiple raters (e.g., experts, observers) when rating multiple items. In general, results are considered acceptable when the mean score exceeds 3.50, and Cv is below 0.25, indicating an acceptable level of consistency in the experts‘ evaluations (Xie et al., 2023; Wang et al., 2021). Kendall's W ranges from 0 to 1 and measures the degree of concordance among multiple ranked variables. Consistency tests of the questionnaire results (Table 2) indicated that, after two rounds of consultation, the experts' evaluations of the indicators exhibited strong consensus and convergence of opinion.

**Table 2:** Coordination coefficient statistics for each level of indicators in the second round of expert opinions.

**Statistic**	**Primary indicator**	**Secondary indicators**	**Tertiary indicators**
Number of indicators	6	15	53
Coordination coefficient (W)	0.726	0.629	0.412
Chi-square value (χ^2^)	43.578	105.646	272.191
Asymptotic significance (p)	0.000	0.000	0.000

### 2.2 Precedence chart method

The Precedence Chart (PC) method, first introduced by P. E. Moody in 1983, was employed to determine indicator weights through pairwise comparisons (Wang and Tian, 2016). In this method, the mean value for each indicator was calculated from expert ratings, and each indicator was compared with others in pairs, with scores of 1, 0.5, or 0 assigned to reflect their relative importance. All pairwise comparison results were compiled into a weight calculation matrix. The TTL value for each indicator was obtained by summing the pairwise comparison scores for that indicator in the matrix. Finally, the weight of each indicator was calculated by dividing its TTL value by the sum of all TTL values, ensuring that the importance of each indicator was accurately reflected in the final system. The method relies on complementary testing, ensuring that each collected questionnaire satisfies the consistency requirement (Li et al., 2023).

### 2.3 Statistical analysis

To analyze and process the data from the two rounds of expert consultation, Excel, SPSS 20.0, and the SPSSAU online data science platform were applied. These tools were used to finalize the indicator system and compute the weights of influencing factors in volleyball coaches' in-game tactical decision-making.

## 3 Construction of the indicator system and results

### 3.1 Conceptual framework and theoretical basis for indicator selection

#### 3.1.1 Conceptual framework for indicator system construction

**Figure 1:**
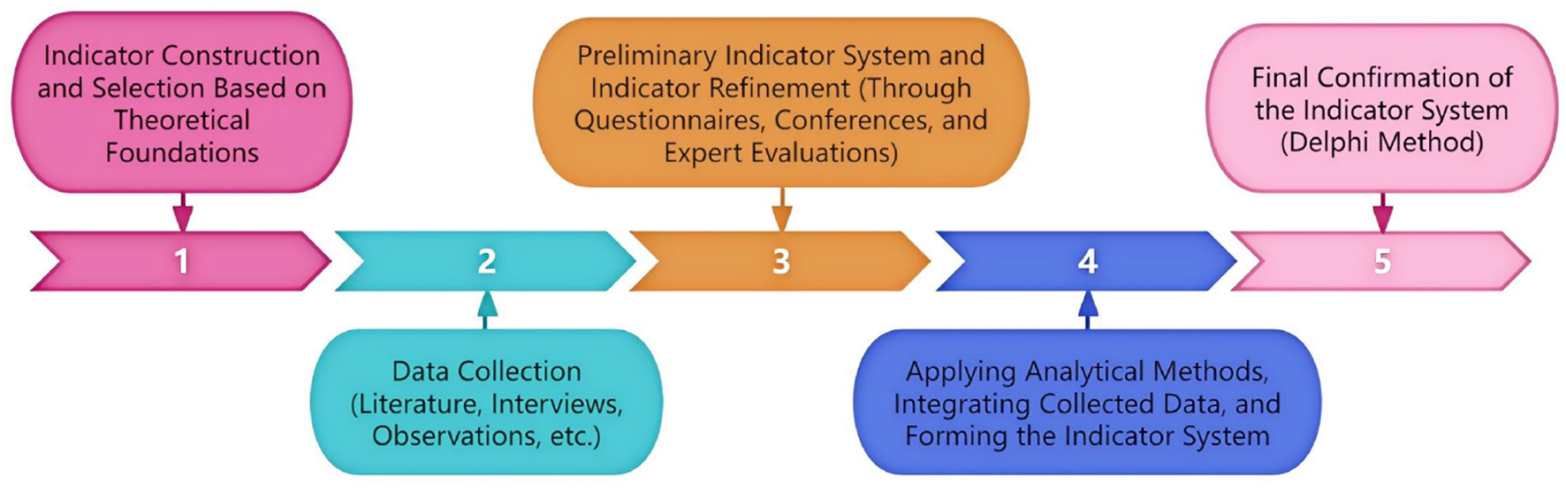
Process of constructing the indicator system.

The development of the indicator system followed a dialectical process of 'concrete–abstract–concrete,' which represents a progressive deepening of understanding of the research object's scope and characteristics. This process involves refinement, improvement, and eventual systematization of knowledge (Hao and Zong, 2007). Building on previous research, the study integrated analytical and synthetic approaches with the Delphi method. By drawing upon competition practice, prior studies, expert judgment, and the researchers' own understanding of influencing factors, an initial set of indicators was established, with coaches' tactical decision-making serving as the focal point. The indicators were then systematically categorized and refined, leading to the development of a rational and coherent system. The construction process can be summarized in five steps: establishing theoretical foundations, collecting data, preliminary indicator selection, indicator screening, and finalizing the indicator system (Figure 1).

#### 3.1.2 Theoretical foundations for indicator selection

Indicators serve as conceptual tools to describe either the overall or specific attributes of a research object. In complex systems, multiple factors coexist, but only those that exhibit strong or significant associations with the object, and that have been refined through systematic processing, can be considered valid indicators.

This study focuses on the guiding question: How do volleyball coaches make in-game tactical decisions? To address this, factors closely related to tactical decision-making were selected, drawing upon three theoretical perspectives: systems theory, sports competition theory, and sports training theory.

1) Systems theory perspective: Volleyball is a complex and dynamic system in which internal components interact in diverse and extensive ways. These interactions evolve over time, creating new challenges. In-game tactical decisions by coaches are made to address these challenges, ensuring the continuity of play or striving for favorable outcomes. Consequently, the structural elements of the system and their characteristics are inherently linked to tactical decision-making.
2) Sports competition theory perspective: Sports competition is a broad concept that includes event management by organizers, refereeing, participation of athletes, coaches, and teams, as well as the involvement of spectators and media. All of these elements inevitably influence coaches' tactical decisions during volleyball matches.
3) Sports training theory perspective: Concepts such as the structure and development of athletes' competitive abilities, determinants of performance, and the notion of athletic potential also play an important role in shaping coaches' in-game tactical decision-making.

### 3.2 Indicator pool and hierarchical structure development

#### 3.2.1 Establishment of the indicator pool

Guided by the conceptual framework and theoretical foundations of the study, both analytical and synthetic methods were employed to identify influencing factors from literature and interview data. Several “meta-indicators” were decomposed or refined during this process. The literature sources included books and journal articles on sports training, competitive performance, coaching practice, and match management.

Indicators were collected through multiple channels: adopting measures from prior studies, transferring relevant indicators from other sports, incorporating expert suggestions, generating new indicators based on the researchers' insights, and extracting items from qualitative interviews. The indicator pool was developed progressively in multiple stages.

After repeated reviews and checks, the collected indicators were consolidated. Duplicated or highly similar items, as well as those with weak relevance, were removed. Some indicators were also renamed for clarity. Ultimately, 119 indicators influencing volleyball coaches' in-game tactical decision-making were identified, forming the foundation of the indicator pool.

#### 3.2.2 Development of the hierarchical indicator structure

In practice, a gradient-based approach was applied to clarify the dominance and inclusion relationships among indicators. This procedure also minimized compatibility issues, reducing redundancy and overlap, thereby ensuring the scientific rigor, systematic coherence, and comprehensiveness of the indicator framework.

**Figure 2:**
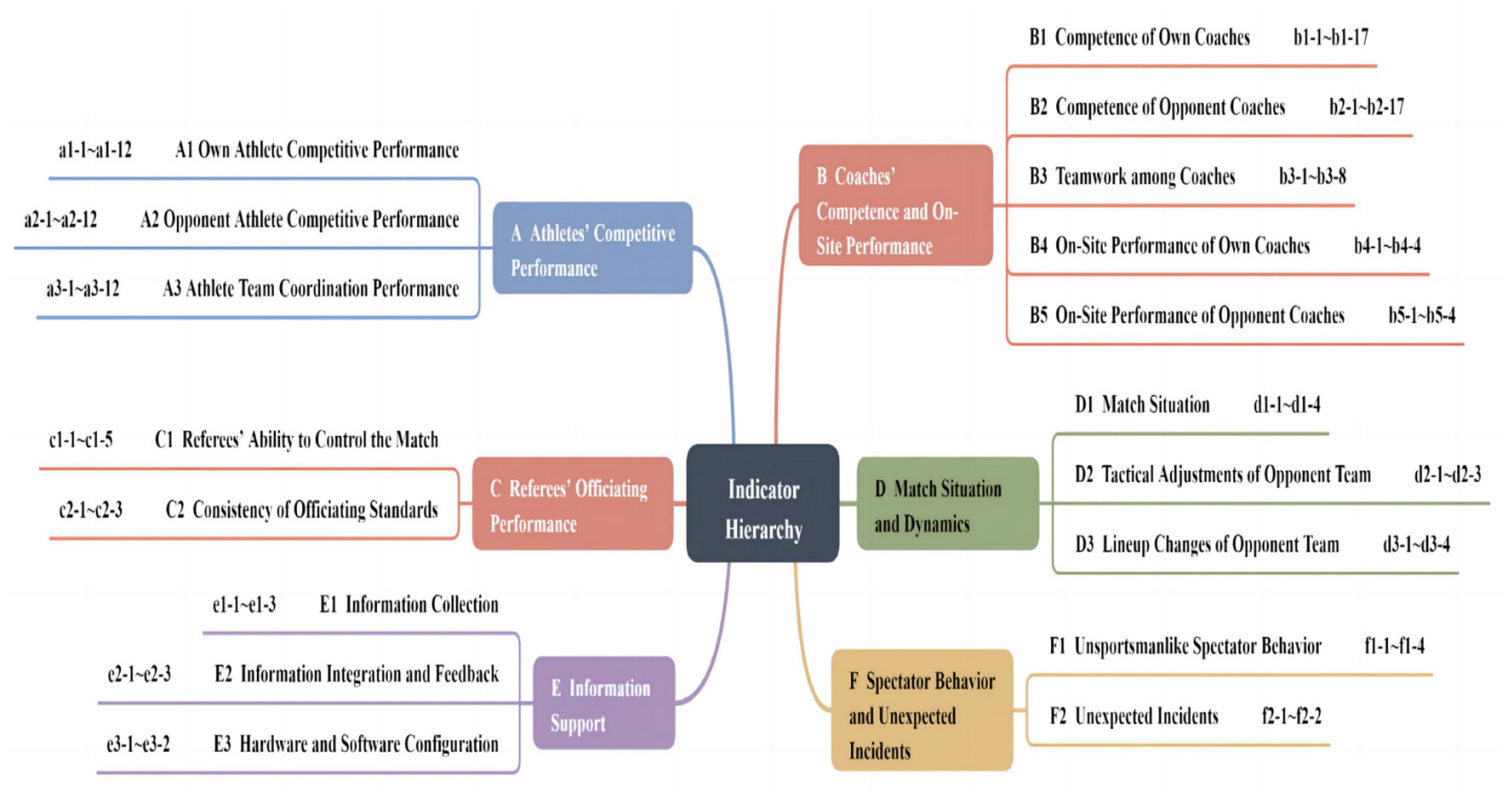
Hierarchical structure of the volleyball coach's in-game tactical decision-making indicator system.

Drawing on the extracted influencing factors and guided by established principles for indicator determination, a synthetic method was employed to consolidate and categorize the indicators. Factors belonging to similar domains or categories were grouped accordingly. Primary indicators were labeled A, B, C, and so forth; for example, secondary indicators were represented as A1, A2, etc., while tertiary indicators were denoted as a1-1, a1-2, etc., derived directly from the indicator pool. Both primary and secondary indicators were explicitly named, leading to the preliminary construction of the hierarchical system (Figure 2). The resulting framework consisted of six primary indicators, eighteen secondary indicators, and one hundred and nineteen tertiary indicators.

### 3.3 Indicator screening

#### 3.3.1 Expert-based preliminary screening

To strengthen the scientific validity and rationality of the indicator system, an expert consultation approach was adopted. The objectives were twofold: (1) to evaluate the structure, content, hierarchical relationships, and clarity of the indicator system; and (2) to conduct an experience-based screening of the indicators.

Five distinguished volleyball scholars and coaches were invited to serve as experts. A preliminary screening questionnaire was distributed via in-person visits, email, and WeChat. The responses were collected, statistically analyzed, and synthesized to guide revisions of the system. Results indicated that the classification, naming, and structural content of the primary, secondary, and tertiary indicators were generally appropriate. Nonetheless, improvements in wording were required, and redundant or less influential indicators needed removal.

Specifically, the experts recommended eliminating the secondary indicator “B2 Opponent Coach Competence” along with its 17 associated tertiary indicators, as these were deemed less relevant to in-game tactical decision-making and of limited influence. At the tertiary level, certain indicators were merged, renamed, or deleted (Table 3). After incorporating expert feedback, the revised indicator system comprised six primary indicators, seventeen secondary indicators, and seventy-nine tertiary indicators.

**Table 3:** Indicators processed based on expert opinions from indicator screening.

**Indicator**	**Suggestions or opinions**	**Resolution**
B2	Delete	Delete
a1-1 was merged with a1-2, a1-10 with a1-11, a2-1 with a2-2, and a2-10 with a2-11	Merge “Individual Technique” and “Individual Tactics” into “Individual Technical and Tactical Performance”	Merge
a2-4, a2-5, a2-6, a2-7, a2-12, a3-7, a3-8, b2-1—b2-17, b3-3, b3-4, b3-5, b3-6, b3-7, b3-8, b4-3, b4-4, b5-3, b5-4, e2-2, f2-4	Similarity with other indicators or weak degree of correlation	Delete

#### 3.3.2 Expert panel discussion screening

After the preliminary screening, three experts were invited for a panel discussion to further refine the indicator content, enhancing the system's operability and accuracy. Based on their recommendations (Table 4), the primary indicators remained unchanged. At the secondary level, “C1 Referees' Ability to Control the Game” was revised to “Refereeing Team Performance.” At the tertiary level, several indicators were merged, renamed, deleted, and reordered.

**Table 4:** Indicators processed based on the group experts' discussion.

**Indicator**	**Suggestions or opinions**	**Resolution**
C1	Revision of indicator names	Revision
a3-3 was merged with a3-4, a3-9 with a3-10, b1-6 with b1-8, b1-9 with b1-10, b1-11 with b1-12, b1-13 with b1-14, d1-2 with d1-3, d2-2 with d2-3, and d3-1 with d3-2	Merge “Overall Tactics” and “Local Tactics”, Merge coaches' understanding of “Officiating Rules” and “Competition Rules”, Merge coaches' “Specialized Techniques” and “Tactics”, Merge coaches' “Understanding” of players with “Trust” in players, Merge coaches' ability to “Filter” information with “Analyze” information, Merge “Critical Nodes” of the match with “Phases” of the match	Merge
a1-9, a2-9, b1-1, c1-3, c1-4, d1-1	Revision of Indicator Names	Revision
a1-4, a1-5, a1-6, a1-7, a1-10, a1-11, a1-12, a2-10, a2-11, b1-4, c1-1, c1-2, c1-5, d3-3	Similarity with other indicators or weak degree of correlation	Delete

As a result, a preliminary indicator system of influencing factors for volleyball coaches' in-game tactical decision-making was established, comprising six primary indicators, seventeen secondary indicators, and fifty-six tertiary indicators.

### 3.4 Finalization of the indicator system

After revisions based on expert feedback and panel discussion screenings, a preliminary indicator system of factors influencing volleyball coaches' in-game tactical decision-making was established. A formal questionnaire was then developed and administered based on this system. Using the Delphi method, the importance of each indicator was assessed, leading to further refinements of the system.

The evaluation process incorporated expert ratings of indicator importance. For each level of indicators, the arithmetic mean (Mj), full-score frequency (Kj), coefficient of variation (Cv), and Kendall's coefficient of concordance (W) were calculated. These measures helped determine the relative importance of indicators and assess the level of consensus among experts.

#### 3.4.1 Revision of primary and secondary indicators

The revision of primary and secondary indicators was guided by the arithmetic mean (Mj) and coefficient of variation (Cv), with thresholds set at Mj ≥ 3.50 and Cv ≤ 0.25. Indicators meeting these criteria were retained, while those falling short were eliminated.

In the first round of consultation, the secondary indicator “B5 Opponent Coach In-Competition Performance” yielded an Mj of 3.35 and a Cv of 0.25, and “E3 Hardware and Software Configuration” recorded an Mj of 3.14 and a Cv of 0.25. Based on these values and expert recommendations, both B5 and E3 were removed. The tertiary indicator “e3-2 Advancement of Analysis Software,” originally under E3, was reassigned to “E2 Information Integration and Feedback.”

In the second round of consultation, after these adjustments, Mj values for primary indicators ranged from 3.75 to 5, while those for secondary indicators ranged from 3.50 to 5. Cv values for primary and secondary indicators ranged from 0 to 0.14 and from 0 to 0.15, respectively. The Kendall's coefficients of concordance (W) were 0.628 and 0.726, with *p*-values of 0.00 (< 0.01). These results indicate a high level of coordination and consistency among expert evaluations, confirming the inclusion of both primary and secondary indicators in the finalized system.

#### 3.4.2 Revision of tertiary indicators

Given the relatively large number of tertiary indicators, the threshold method was adopted for their refinement. Thresholds were calculated for each indicator based on full-score frequency, mean value, and coefficient of variation (Table 5). Indicators with comparatively low importance were excluded accordingly.

**Table 5:** Threshold table from two rounds of expert consultation.

**Statistic**		**Mean**	**Standard deviation (SD)**	**Threshold**
Results of the first round of expert consultation	FSF	0.5729	0.2315	0.3414
	M	4.50	0.31	4.19
	CV	0.1257	0.0434	0.1692
Results of the second round of expert consultation	FSF	0.5879	0.2409	0.3470
	M	4.56	0.028	4.28
	CV	0.1122	0.0363	0.1485

The thresholds were computed using the following formulas: for full-score frequency and mean values, Threshold = Mean – Standard Deviation; for the coefficient of variation, Threshold = Mean + Standard Deviation. To minimize the risk of discarding critical indicators, inclusion in the final system required that an indicator simultaneously satisfy the following criteria: (a) full-score frequency and mean values exceeding the threshold, and (b) coefficient of variation below the threshold. Indicators failing to meet these criteria were removed.

Following the established screening criteria, the first round of expert ratings led to the elimination of indicators with a full-score frequency below 0.34, a mean score under 4.19, and a coefficient of variation exceeding 0.17. After expert consultation, three indicators—b5-1 Changes in Opponent Coach Behavior, b5-2 Changes in Opponent Coach Psychology, and e3-1 Advancement of Communication Equipment—were removed.

In the second round, indicators with a full-score frequency below 0.35, a mean score under 4.28, and a coefficient of variation above 0.15 were excluded. Only one indicator, F1-2 Spectator Verbal Abuse, met these criteria and was removed.

After screening and reordering, the finalized indicator system for factors influencing volleyball coaches' in-game tactical decision-making was established. The system included six primary indicators, fifteen secondary indicators, and fifty-two tertiary indicators (**Table 7**).

### 3.5 Determination of indicator weights

This study employed a combined approach using the Delphi method and the Precedence Chart technique to determine the weights of indicators at each level, aiming to reveal the influence of each indicator on volleyball coaches' in-game tactical decision-making.

The Precedence Chart method is a widely used weighting technique that involves constructing a weight calculation matrix to compare relative importance and compute the weights. Following the basic paradigm of the method, the mean value of each indicator was calculated based on expert ratings. Pairwise comparisons were then conducted according to the mean values to build the Precedence Chart weight calculation matrix (Table 6). Subsequently, the scores of each indicator were summed horizontally to obtain their relative values. After normalization, the weight of each indicator was derived (Table 7).

**Table 6:** Weight calculation table for primary indicators based on priority diagram.

**Mean**	**Item**	**A. Athlete's competitive performance**	**B. Coach's competence and on-site performance**	**C. Referee's officiating performance**	**D. Match situation and dynamics**	**E. Information support**	**F. Spectator behavior and unexpected incidents**
5	A. Athlete's competitive performance	0.5	1	1	1	1	1
4.917	B. Coach's competence and on-site Performance	0	0.5	1	1	1	1
3.833	C. Referee's officiating performance	0	0	0.5	0	0	0.5
4.667	D. Match situation and dynamics	0	0	1	0.5	1	1
4.25	E. Information support	0	0	1	0	0.5	1
3.833	F. Spectator behavior and unexpected incidents	0	0	0.5	0	0	0.5

A value of 0 indicates relatively unimportant, 1 indicates relatively more important, and 0.5 indicates equally important.

**Table 7:** Indicator system of factors influencing volleyball coaches' in-game tactical decisions and their indicator weights.

**Primary indicator**	**Secondary indicator**	**Tertiary indicator**
A Athlete competitive performance (0.3056)	A1 Own athlete competitive performance (0.1067)	a1-1 Individual technical and tactical performance of own players (0.0381)
		a1-2 Physical condition changes of own players (0.0314)
		a1-3 Self-adjustment status of own players (0.0270)
		a1-4 Psychological changes of own players (0.0222)
	A2 Opponent athlete competitive performance (0.1067)	a2-1 Individual technical and tactical performance of opposing players (0.0314)
		a2-2 Physical condition changes of opposing players (0.0148)
		a2-3 Psychological changes of opposing players (0.0056)
		a2-4 Self-adjustment status of opposing players (0.0056)
	A3 Athlete team coordination performance (0.1200)	a3-1 On-court communication of own players (0.0181)
		a3-2 Changes in mutual trust among own players (0.0314)
		a3-3 Degree of tactical execution by own players (0.0359)
		a3-4 Performance of lineup combination strength of own team (0.0359)
		a3-5 Degree of tacit cooperation among own players (0.0314)
		a3-6 Degree of tactical execution by opposing players (0.0222)
		a3-7 Degree of tacit cooperation among opposing players (0.0115)
		a3-8 Performance of lineup combination strength of opposing team (0.0222)
B Coach competence & in-competition performance (0.2500)	B1 Own coach competence (0.1289)	b1-1 Coaches' professional knowledge reserves (0.0270)
		b1-2 Coaches' decision-making style (0.0270)
		b1-3 Coaches' tactical style (0.0270)
		b1-4 Coaches' personality and psychological traits (0.0085)
		b1-5 Coaches' understanding of rules and officiating (0.0222)
		b1-6 Coaches' understanding of competition patterns (0.0314)
		b1-7 Coaches' knowledge of and trust in players (0.0359)
		b1-8 Coaches' specialized technical and tactical competence (0.0181)
		b1-9 Coaches' information-gathering ability (0.0314)
		b1-10 Coaches' authority and prestige (0.0359)
		b1-11 Coaches' emotional regulation ability (0.0181)
		b1-12 Coaches' competition experience in leading teams (0.0222)
	B2 Coaching staff collaboration (0.0489)	b2-1 Degree of communication within the coaching team (0.0148)
		b2-2 Degree of cooperation within the coaching team (0.0270)
	B3 Coach's in-competition performance (0.0400)	b3-1 Behavioral changes of coaches (0.0148)
		b3-2 Psychological changes of coaches (0.0115)
C Officiating performance (0.0566)	C1 Officiating team performance (0.0267)	c1-1 Degree of cooperation within the referee team (0.0004)
		c1-2 Degree of communication within the referee team (0.0022)
	C2 Officiating standard (0.0267)	c2-1 Strictness of referees' foul decisions (0.0041)
		c2-2 Consistency of referees' officiating standards (0.0085)
D Match situation & dynamics (0.1944)	D1 Match situation (0.0933)	d1-1 Changes in match tempo (0.0222)
		d1-2 Critical moments and phases of the match (0.0222)
		d1-3 Score situation (0.0314)
	D2 Opponent lineup & changes (0.0578)	d2-1 Opponent's starting lineup (0.0085)
		d2-2 Changes in opponent's on-court lineup (0.0222)
	D3 Opponent tactical changes (0.0667)	d3-1 Changes in opponent's tactical system (0.0359)
		d3-2 Changes in opponent's individual tactical patterns (0.0148)
E Information support (0.1389)	E1 Information collection (0.0844)	e1-1 Degree of data collection on own players' technical and tactical indicators (0.0085)
		e1-2 Degree of data collection on opponent players' technical and tactical indicators (0.0085)
	E2 Information integration & feedback (0.0756)	e2-1 Effectiveness of key information capture (0.0148)
		e2-2 Efficiency of information feedback (0.0115)
		e2-3 Advancement level of analytical software (0.0056)
F Spectator behavior & incidents (0.0566)	F1 Unruly spectator behavior (0.0044)	f1-1 Audience noise interference (0.0011)
		f1-3 Audience disruption of match progress (0.0022)
	F2 Incidents (0.0133)	f2-1 Player injuries during the match (0.0148)
		f2-2 Various conflicts occurring during the match (0.0033)

## 4 Analysis and discussion

### 4.1 Athlete competitive performance

The athletic performance of athletes received the highest weight coefficient (0.3056), highlighting its decisive role in volleyball coaches' in-game tactical decision-making. The core driving force of a volleyball match lies in the confrontation between athletes on the court, while other system elements are shaped by this core interaction. In the specific match process, athletes' performance serves as the source of tactical decision-making issues for the coach, and the coach's tactical decisions directly influence the athletes' performance. The two are mutually causal and interdependent. From the perspective of the factors determining match outcomes, the performance of both the home and opponent athletes on the court is the core factor affecting the match result. From the perspective of tactical decision content, the performance of the athletes and teams on both sides is the primary source of the decision-making issues. Generally speaking, excellent coaches can clearly define the decision-making problem based on both their own and the opponent's athletes' individual or team performance, weigh various options, and make relatively ideal tactical decisions (Costa et al., 2018; Vergeer and Lyle, 2009).

Within this dimension, A3 Team Coordination Performance (0.1200) carried the highest weight among the three secondary indicators. Team coordination, which represents the essence of collective strength, is a decisive factor in shaping match momentum and outcomes. Unlike many other team sports, volleyball places greater demands on cooperation and collective commitment due to its restrictive rules of play. A common issue in team sports is the “team paradox,” whereby players, despite sharing common goals and prearranged tactics, may fail to coordinate effectively during competition—exemplifying the phenomenon of “unity without synergy” (Zheng, 1987). From a game theory perspective, athletes act as rational individuals seeking personal utility maximization rather than collective benefit. However, according to team reasoning theory (Colman et al., 2008), the structural constraints of volleyball—such as the three-hit rule and the prohibition of consecutive contacts—along with the high level of interdependence among teammates, suggest that optimal outcomes for both the team and individuals can only be achieved by integrating collective and individual actions. In this context, volleyball athletes are more likely to prioritize cooperation and commitment, thereby balancing individual and collective rationality. For coaches, the collective performance of athletes holds greater strategic significance than isolated individual abilities and exerts a stronger influence on in-game tactical decision-making.

### 4.2 Coaches' competence and on-site performance

Coaches' competence and on-site performance ranked second (weight coefficient = 0.2500), emphasizing the critical role of the coach's personal qualities and decision-making ability in shaping tactical decisions during a match. Volleyball matches require coaches to respond rapidly to highly dynamic and uncertain environments, adjusting tactics in real-time to align with strategic goals. The scientific and rational nature of these decisions often determines whether a team can win the match. Effective in-game decision-making requires coaches to possess a broad range of skills, including strong theoretical knowledge, practical experience, psychological resilience, authority, keen situational awareness, information processing abilities, and teamwork skills. Previous studies have shown that coaches with excellent coaching abilities who communicate effectively and positively with athletes can enhance team cohesion and organizational behavior, thereby having a positive impact on athletic performance (Oh, 2023). Additionally, coaches' leadership behaviors are key factors influencing athletes' psychological resilience and competitive performance (Wang, 2023), and democratic leadership behaviors in coaches are positively correlated with athlete performance and team effectiveness (Liu et al., 2025). Furthermore, a coach's authority, credibility, or leadership influence is indeed linked to athletic performance and team outcomes, as reflected in athletes' compliance with the coach and tactical execution (Rylander, 2016).

As commanders and decision-makers, volleyball coaches also experience psychological and behavioral fluctuations during the dynamic changes of a match. In situations requiring rapid tactical adjustments, excessive cognitive load and increased psychological stress may lead to suboptimal decisions (Fletcher and Scott, 2010). In contrast, experienced coaches, with enhanced cognitive abilities and deeper knowledge, are often able to make more accurate judgments of the match situation and athlete performance, allowing for more flexible and adaptive resource allocation and tactical strategy implementation (Vergeer and Lyle, 2009; Roca and Gomes, 2022; Raab, 2003). From this perspective, a volleyball match is not only a contest of athletic performance but also a rigorous test of the coach's abilities and decision-making skills.

### 4.3 Match situation and dynamics

The weight coefficient for the “Match Situation and Dynamics” indicator is 0.1944, ranking third, which highlights the importance of closely monitoring and addressing match circumstances and dynamics in volleyball coaches' in-game tactical decision-making. The match situation and its changes not only serve as the source of tactical decision-making issues but also provide the essential foundation for tactical adjustments. Research has shown that team tactical behaviors fluctuate in phases depending on the match status and team ranking context, further emphasizing the critical role of situational dynamics in shaping in-game tactical decisions (Ramos et al., 2017).

In competitive volleyball, significant shifts in score margins and momentum are often described metaphorically as “rising” and “falling.” When one team gains a substantial lead, the momentum is described as “surging” or “ebbing,” while closely contested points are characterized as “in equilibrium” (Chen and Tian, 2013). These descriptions vividly capture the evolving dynamics of the match. “Situation” reflects the state of competition at a specific point in the match, whereas “dynamics” represent the inherent patterns of play, illustrating the natural flow of the game.

Typically, when the match rhythm is disrupted, during critical phases, or when a large score gap appears, coaches make tactical decisions such as substitutions, time-outs, or adjustments to the game plan. Substitutions can alter the tactical system, strengthen either the offensive or defensive lines, and must be adjusted flexibly based on the match conditions to optimize performance (Mozolev et al., 2023). Time-outs provide players with recovery opportunities, facilitate tactical redirection, and can disrupt the opponent's momentum, helping stabilize the team's performance (Fernández-Echeverria et al., 2013). Research indicates that coaches are more likely to call a time-out when the score difference is between 1 and 4 points (López-Serrano and Martín, 2025); notably, 63.3% of time-outs are called while trailing, and 50.28% of those lead to winning the subsequent rally (Zetou et al., 2008).

In the highly competitive nature of volleyball, both teams aim to maximize their strengths while simultaneously neutralizing their opponents' advantages. This ongoing interaction of constraint and counter-constraint permeates the match. From this perspective, in-game tactical decision-making can be seen as a dynamic process where coaches continuously adjust line-ups and strategies based on evolving match situations and fluctuations.

### 4.4 Information support

Information Support ranked fourth among all primary indicators (weight coefficient = 0.1389), highlighting its importance in in-game tactical decision-making. Decisions inherently depend on information; while pre-match data collection is relatively static, in-game information is dynamic and continuously evolves as the match progresses, reflecting the interactive relationships among elements of the competitive system. To address this challenge, coaches and their support teams must allocate responsibilities effectively, collaborate efficiently, and carefully interpret the unfolding game to gather multidimensional real-time data on both their own and the opposing team's performance. Match analysis remains a key method for extracting valuable insights from competition (Fernandez-Echeverria et al., 2017). With the rapid advancement of artificial intelligence, algorithms, and big data technologies, the capacity of coaches and researchers to collect and process information in real time has been significantly enhanced (McGillick et al., 2024). Nonetheless, three essential attributes—reliability, completeness, and accuracy—must be ensured in the collection of decision-related information (Wang and Qin, 2010). Reliability concerns the authenticity and validity of data, requiring careful refinement and interpretation by coaches. Completeness reflects the breadth of information obtained, depending on effective collaboration within the coaching team. Accuracy pertains to the granularity of information and its ability to capture event-specific details, thereby reducing uncertainty in decision-making.

Ultimately, the quality of tactical decisions during competition is strongly determined by the quality of the information collected, which should reflect the objective reality of the match. While different decisions may demand varying levels of informational precision, coaches must remain aware of the inherent limitations of the available data. In general, however, the more reliable, complete, and accurate the information, the more rational and effective the tactical decisions are likely to be.

### 4.5 Officiating performance

Officiating Performance carried a weight coefficient of 0.0566, equal to Spectator Behavior and Incidents, and ranked fifth among the primary indicators. This factor reflects the refereeing team's capacity to manage the match, particularly in terms of teamwork and consistency of officiating standards. Referees are required not only to judge fouls with fairness and justification but also to maintain uniformity in enforcement while balancing the flow of the game to ensure transparency and impartiality.

Empirical evidence suggests that psychological qualities such as confidence, decisiveness, courage, and resilience play a crucial role in referees' officiating performance (Diotaiuti et al., 2020). Although officiating experience is positively associated with decision-making ability (Catteeuw et al., 2009), effective refereeing depends on more than individual expertise. It requires strong collaboration and communication within the officiating team, as well as rationality and consistency in judgment on the court. Effective teamwork, timely communication, and the use of advanced technological tools are essential for ensuring the orderly conduct of modern volleyball competitions. Studies have further shown that referees who maintain good communication with other match participants report higher self-efficacy, which indirectly enhances officiating quality (Diotaiuti et al., 2017). Moreover, the ability to apply rules accurately in critical moments is vital (American Sport Education Program, 2007), as timely and precise decisions regarding fouls can shape match momentum, determine outcomes, or become focal points of controversy.

Although Officiating Performance ranked relatively low compared to other indicators, its influence on coaches' in-game tactical decisions and overall match outcomes remains significant. In critical phases or at decisive score points, refereeing decisions may directly trigger coaches' tactical adjustments or behavioral responses.

### 4.6 Spectator behavior and unexpected incidents

Spectator Behavior and Unexpected Incidents carried a weight coefficient of 0.0566. Although relatively low, this factor can significantly disrupt match processes and influence coaches' in-game tactical decisions. While watching sports is considered a civilized form of leisure and an integral part of cultural life, spectators often exhibit strong emotional biases. Support is typically directed toward home teams or favored players, whereas opponents are subjected to booing or hostile reactions, a phenomenon described in sports culture as the “bias theory” (Zhang et al., 2018). Evidence indicates that spectator behavior can affect the physiological and psychological states of athletes, referees, and coaches, thereby shaping their performance and, in some cases, determining match outcomes (Oldfield et al., 2024).

Spectator influence is most commonly expressed through noise interference and disruption of order. Noise includes shouting, booing, chanting, verbal abuse, and artificial sounds from instruments, all of which may disturb athletes' focus and the rhythm of play. Research indicates that crowd noise alters the physiological and psychological responses of both home and visiting players (Greer, 1983), and can even bias referees' decisions (Nevill et al., 2002). At critical moments, or when players experience emotional setbacks, such disturbances may escalate into conflicts. Emotional loss of control among spectators may lead to abusive language, object throwing, clashes, or even physical assaults on referees, coaches, or athletes. Neurological studies suggest that such behaviors may partly arise from the activation of mirror neurons in motor-related cortical areas during spectatorship (Beilock et al., 2008).

For coaches, preventive strategies are essential. Teams should establish discipline, regulate athletes' conduct, and prepare contingency plans before competition. During incidents, coaches must remain composed, manage athletes' emotions, and minimize harm caused by disruptive behavior. Additionally, injuries to athletes represent another major form of disruption, particularly when they involve key players. Such injuries may compromise tactical systems and weaken overall performance (Surya et al., 2015). In these cases, coaches should promptly involve medical staff and adopt tactical measures such as substitutions or time-outs to reduce the negative impact on team competitiveness.

## 5 Conclusion

This study aimed to identify the key factors that influence volleyball coaches' tactical decision-making during matches and to evaluate their relative importance. Building on systems theory, training theory, and competition theory, we integrated prior research with the practical insights of experienced domestic coaches to establish a structured indicator framework. The final system, consisting of six primary indicators, fifteen secondary indicators, and fifty-two tertiary indicators, provides a comprehensive reference for understanding the complexity of in-game decision-making. Additionally, it offers coaches a practical tool to analyze match situations, design targeted training plans, and support evidence-based tactical decisions in dynamic competition settings. More importantly, the system provides valuable learning resources for novice volleyball coaches and those aspiring to pursue a career in volleyball coaching, helping them learn and understand the complexity and key factors of tactical decision-making, and apply it effectively in practice.

In actual matches, coaches can use the indicator system to make tactical adjustments, time-out decisions, and substitutions based on the specific circumstances. For example, when the game tempo is unbalanced or the opponent's offense is too strong, coaches can use indicators such as “game tempo” and “opponent tactics” to quickly adjust defensive strategies or substitute players with lower energy to restore balance between offense and defense. When trailing in score, coaches can identify players showing signs of fatigue, technical errors, or emotional instability through the “player performance” indicator. This allows them to adjust the lineup, intensify the offense, or quickly substitute key players to strengthen the offensive system. Additionally, coaches can adjust the pace of the game using the “game tempo” indicator, using time-outs to disrupt the opponent's offensive rhythm, restore their own team's playing state, and seize the opportunity to overtake.

Nonetheless, this study has certain limitations. The expert panel was relatively homogeneous, consisting primarily of male coaches and scholars from China, without the inclusion of female or international experts. This limitation restricts the generalizability of the results across different cultural and gender contexts. Future research should expand the diversity of the expert panel, incorporating experts from diverse backgrounds, including female coaches and international perspectives, to ensure the framework's applicability in different volleyball systems globally. Furthermore, the indicator weights were derived primarily from expert judgments and were not validated with real match data. This reflects the inherent challenge of systematically capturing and quantifying coaches' in-game tactical decisions. Future studies should incorporate real match data and performance outcomes to empirically validate and refine the framework. This will not only enhance the practical relevance of the system but also provide data-driven insights into the relationship between tactical decision-making and match performance.

Another promising direction for future research is the development of intelligent decision-support systems. These systems could integrate big data, artificial intelligence, and real-time match analysis to assist coaches in optimizing tactical adjustments during matches. Such systems could help reduce decision-making uncertainty, improve adaptability, and ultimately enhance competitive performance. By bridging theoretical development with practical application, these efforts will provide coaches with more precise, adaptive, and context-sensitive tools to manage the complexities of in-game tactical decisions. Additionally, future research should explore the integration of cross-sport methodologies to enhance the robustness of the framework and expand its applicability to other team sports, ultimately creating a more universal tool for tactical decision-making across various sports disciplines.

## Data Availability

The original contributions presented in the study are included in the article/supplementary material, further inquiries can be directed to the corresponding authors.
